# A New Strategy to Increase Production of Genoprotective Bioactive Molecules from Cotyledon-Derived *Silybum marianum* L. Callus

**DOI:** 10.3390/genes11070791

**Published:** 2020-07-14

**Authors:** Dina Gad, Mahmoud Elhaak, Andrea Pompa, Magdy Mattar, Mohamed Zayed, Daniele Fraternale, Karl-Josef Dietz

**Affiliations:** 1 Biochemistry and Physiology of Plants, Faculty of Biology W5, Bielefeld University, 33501 Bielefeld, Germany; dinagad@ymail.com; 2 Faculty of Science, Botany Department, Menoufia University, Shebin EL-koum, Egypt; mmattar62@yahoo.com (M.M.); mzayed48@yahoo.com (M.Z.); 3 Faculty of Science, Botany Department, Tanta University, Tanta, Egypt; abdelhaakmah@yahoo.com; 4 Department of Biomolecular Sciences, University of Urbino “Carlo Bo” Via Donato Bramante, 28, 61029 Urbino (PU), Italy; andrea.pompa@uniurb.it

**Keywords:** *Silybum marianum*, silymarin, asparagine, 6-benzylaminopurine, callus induction, chlorogenic acid, 3,5-*O*-dicaffeoylquinic acid, genotoxicity, Ames test

## Abstract

There is a need to enhance the production of bioactive secondary metabolites and to establish new production systems, e.g., for liver-protective compounds of *Silybum marianum* seeds. Quantifying and identifying the produced phytochemicals, and examining their protective effects against genotoxic agents, is of great interest. This study established a protocol for the qualitative and quantitative production of hepatoprotective compounds in cotyledon-derived *Silybum marianum* callus through optimized supplementation of the MS medium with the growth regulators 2,4-D, benzylaminopurine, myoinositol, and asparagine. High-performance liquid chromatography (HPLC) coupled with electrospray ionisation mass spectrometry (ESI-MS) allowed for identification and quantification of the produced compounds. None of the growth medium combinations supported a detectable production of silymarin. Instead, the generated calli accumulated phenolic acids, in particular chlorogenic acid and dicaffeoylquinic acid, as revealed by HPLC and mass spectrometric analysis. 4-Nitro-o-phenylenediamine (NPD) was employed in the AMES-test with *Salmonella typhimurium* strain TA98 because it is a potent mutagen for this strain. Results revealed that callus extract had a high anti-genotoxic activity with respect to standard silymarin but more evident with respect seed extract. The callus produced chlorogenic acid and dicaffeoylquinic acid, which revealed higher bioactivity than silymarin. Both compounds were not formed or could not be detected in the seeds of *Silybum marianum* Egyptian ecotype.

## 1. Introduction

*Silybum marianum* L. Gaertn (Asteraceae) is an annual to biennial herb originating from the Mediterranean and North African regions [1]. Currently it grows wild throughout Europe, North Africa, the Americas, and Australia [2]. The flower heads of the plant are mostly purple or white. *S. marianum* plants flower from June to August in the Northern or December to February in the Southern Hemisphere. *S. marianum* fruits vary from white to nearly black [3]. The medicinal part of the plant is the seeds [4].

*Silybum marianum*, commonly known as milk thistle, is one of the most important and best-researched plants of old times for the treatment of liver and gallbladder disorder, including hepatitis, cirrhosis, jaundice, and supportive therapy against *Amanita phalloides* mushroom intoxication and other toxins [5]. *S. marianum* fruits extract has been used for centuries as a “liver tonic” and is well known to prevent or reverse hepatotoxicity of reactive drug metabolites or naturally occurring toxins [3]. The main active compounds of milk thistle are silymarin and phenolic acids [6,7]. Silymarin, the active component of this plant, is a standardized extract comprised of roughly 70 to 80% silymarin flavonolignans (silybin A and B, isosilybin A and B, silydianin, and silychristin) and flavonoids (taxifolin and quercetin), and the remaining 20–30% are comprised of polymeric and oxidized polyphenolic compounds [8]. Silymarin has a wide range of biological and pharmacological effects including antioxidant activity [9]. Silymarin stimulates protein synthesis and cell regeneration in liver cells making it helpful in treating toxic liver damage, chronic inflammatory liver diseases, and liver cirrhosis [10,11]. It is also reported to exert anticancer activity against some human carcinoma cell lines [12,13]. Plant tissue culture has been developed as a powerful method for in vitro production of plant secondary metabolites under controlled conditions independent on environmental fluctuations [14,15]. Manipulation of culture medium composition can substantially increase the production of secondary compounds [16,17]. Major limitations of *S. marianum* cell cultures come from their instability during long-term culture and low product yield [15]. In this respect, tissue culture systems of *S. marianum* have been established using cotyledon explants [18]. Optimization is particularly useful when the precursors are expensive. In this respect, several products were found to accumulate in cultured cells to a higher level than in native plants through optimized cultural conditions. 

Most secondary metabolites are derived from wild or cultivated plants, as their chemical synthesis is either extremely difficult or economically impossible [19]. *Silybum marianum* plants contain flavonoids, phenols, and tannins, while saponins and alkaloids are not detected. Contents of phenol and tannins are noted very less as compared to flavonoid i.e., 0.430% phenol is recorded in blue flowering and 0.4321% in the white flowering plant [20]. Callus cultures of *S. marianum* are widely known to produce saponins [21]. Plant extracts of saponins exhibit specific pharmacological activities to control many diseases, including hepatoprotective activity [22,23]. The protective activity of the total saponins from *Actinidia valvata* Dunn root (TSAV) was studied against carbon-tetrachloride- (CCl_4_-) induced acute liver injury in mice [24]. Total saponins of *A. valvata* Dunn root are known to be important pharmacologically active constituents against inflammation and tumor [25,26]. 

In vitro plant cultures can be used as natural sources of phenolic compounds for a sustainable use. Unlike plant tissues, the concentration and individual profile of phenolic compounds can be regulated in plant cultures leads to a standardized product [27,28]. Phenolic acids in fruits, vegetables, and other plants have been identified repeatedly as natural antioxidants. Chlorogenic acid (CGA) is a type of polyphenol which widely distributed in plants, but few commercial standards are available, therefore precise identification of individual CGA in complex mixtures is difficult. However, it is possible to differentiate between each isomer on the basis of its fragmentation patterns and chromatographic resolution of a reversed phase packing [29]. CGA exhibits pharmacological activities against inflammation, diabetes, tumors and ulcers [30,31]. 

As mentioned above silymarin exhibits diverse beneficial properties such as preventing or decelerating cancer development, counteracting apoptosis, and as antioxidant or hepatoprotectant [32]. Interestingly, the first reports also describe the anti-genotoxic properties of silymarin. The identification of substances that cause or protect from genotoxicity is of significant general interest [33]. Thus, the chemo-protective activity of silymarin is of high significance and more detailed investigations are highly recommended. Plants are a promising source for secondary metabolites with anti-mutagenic activity. Such anti-mutagens help, for example, by stimulating the cell defense against environmental mutagens. An increasing interest is directed to natural compounds that counteract diseases [34]. Various classes of secondary metabolites are present in the plants and exhibiting anti-mutagenic activities.

The *Salmonella typhimurium* revertant assay (*Salmonella* test; Ames test) is a widely accepted short-term bacterial assay for identifying substances with mutagenic activity. The Ames test is a mutagenesis test based on the detection of DNA mutations in a bacterium that is histidine-dependent. The used strain of *Salmonella typhimurium* TA 98 is auxotroph for histidine (*His*−) and can revert spontaneously to *His+*, and thus grow in a histidine-free medium. The very low spontaneous reversion rate can be increased by mutagens, which allows the mutagenic potential quantification of these substances. Such mutated cells can grow in the absence of histidine and form colonies. Therefore, the test is often referred to as “reversion assay”. The Salmonella mutagenicity test was particularly intended to identify chemically affected mutagenesis [35]. The test is utilized worldwide as an initial screen to evaluate the mutagenic capability of new chemicals, medications and environmental mutagens [36]. 

The aims of this study are to establish a protocol for high yield production of secondary metabolites that have medical importance for liver protection in cultured tissues of *S. marianum*. It was sought to compare the anti-genotoxic and chemo-protective effects of standard silymarin, silymarin extracted from *Silybum marianum* seeds and compounds produced by *S. marianum* cotyledons callus.

## 2. Material and Methods 

### 2.1. Callus Culture

*Silybum marianum* seeds were collected from purple flower plants naturally growing in a neglected area in Gharbiya district (Nile Delta of Egypt) during the 2013–2014 growing season. Seeds were washed several times with distilled water. Following seed coat removal, the two cotyledons were separated from each other, and disinfected prior to starting the in vitro cultures. All steps took place in a laminar flow chamber. Sterilized cotyledons were cultured on a solidified Murashige and Skoog (MS) basal medium. Solidified basal MS media were supplemented with different concentrations of 2,4-D, Asn, BAP, and inositol producing 16 types of media labelled with alphabetical letters from A through O (Table 1). All cultures were incubated in the growth chamber at 25±2 °C in darkness for 2 months during which callus was transferred to a new medium after 21 d interval (at 21, 42, and 63 d). Then the harvested callus were dried at 40 °C, powdered and kept in plastic cups in a refrigerator.

### 2.2. Callus Extract for Chemical Analysis

*Silybum marianum* callus were collected at 63 day for biochemical analysis and dried samples were extracted with ethanol for spectrophotometric quantification of total flavonoids, phenolic compounds, and saponin. For HPLC and MS analysis, dried residue was dissolved in methanol (1 mL) and stored at 4 °C in the dark. Extracted samples were filtered before use for HPLC analysis. 

### 2.3. Estimation of Flavonoids

The modified colorimetric method with aluminum chloride [37] was used to estimate the flavonoid content in callus extracts. A dried callus sample (100 mg) was extracted with 80% ethanol. A defined volume (0.5 mL) was mixed with 1.5 mL of 95% (*v*/*v*) ethanol, 0.1 mL of 10% (*w*/*v*) AlCl_3_, 0.1 mL of 1 M K-acetate and 2.8 mL water. After incubation at room temperature for 30 min, the absorbance was measured at 415 nm with a spectrophotometer (Cary 300, version 9, Agilent, Santa Clara, CA, USA). The same volume of water substituted for the AlCl3-solution in the blank. Quercetin was used as standard for calibration. Flavonoid content was calculated as mg quercetin equivalents/g dry weight (DW) callus. 

### 2.4. Estimation of Saponin

The saponin content was quantified in callus powder; 100 mg was extracted with 85% ethanol (*v*/*v*). The clear supernatants were combined and adjusted to a defined volume. 0.1 mL of ethanol extract was diluted by adding 0.4 mL ethanol 85% (*v*/*v*) and then mixed with 0.5 mL of 8% (*w*/*v*) vanillin in ethanol. The solution was placed in an ice bath and mixed with 5 mL of 72% sulfuric acid, then heated to 60 °C for 10 min followed by cooling in ice-cold water. The absorbance of the formed color was measured at 544 nm. The standard curve was obtained with cholesterol. Saponin content is given as mg cholesterol equivalents/g DW callus [38]. 

### 2.5. Total Phenolic Compounds

Dry callus (100 mg) was extracted with 85% (*v*/*v*) ethanol. Ethanol extract (0.1 mL) was diluted up to 1 mL with distilled water and mixed with 0.1 mL Folin–Ciocalteu reagent and 1 mL Na_2_CO_3_ (20% *w*/*v*), and adjusted to 5 mL with distilled water. Absorbance was measured spectrophotometrically at 650 nm after exactly 30 min. A standard curve was constructed using pyrogallol. Total phenolic compounds content is given in mg pyrogallol equivalents/g DW callus [39].

### 2.6. HPLC Analysis

Silymarin standard solution was prepared from standardized silymarin (Sigma, Germany) by dissolving it in methanol to a concentration of 1 mg/mL. For chromatography, 20 μL of this standard solution or the callus-derived solutions were injected into the HPLC system Lichro Cart at a column temperature of 40 °C, using an RP-18 column (4 × 250 mm, 5 μm) (Merck, D-6100 Darmstadt, Germany). Gradient elution was applied at a flow rate of 1 mL min^−1^. The mobile phase A consisted of water containing 0.1% formic acid, while methanol was used as mobile phase B. The gradient condition was as follows: start 25% A and 75% B, 0–39 min linear change to 55% A and 45% B, 39–40 min linear change to 25% A and 75% B. Phenolic acids were detected at 288 nm.

### 2.7. Mass Spectrometry

The ESI-MS and MS/MS analysis were performed with an Esquire 3000 plus (Bruker Daltonics, Germany) using an ESI source with negative ion mode. The spray voltage was set to 4000 V. The temperature of the drying gas (N_2_) was kept at 180 ºC, the fractions of HPLC peaks were collected and evaporated using vacuum centrifuge, the residue was dissolved in MeOH (50% v/v) and the injection volume was 150 μL.

### 2.8. Bacterial Reverse Mutation Test (Ames Test)

The mutagenicity of NPD was determined in a bacterial reverse mutation assay using *S. typhimurium* tester strain TA98. Silymarin (SIGMA, Deisenhofen, Germany), callus and silybum marianum seed extracts were tested for protection against genotoxicity of NPD by the pre-incubation method [40]. Briefly, dry powders of callus and seeds (200 mg) were defatted using petroleum ether and extracted with 95% ethanol. Ethanol was evaporated at room temperature under a hood, and the extracted yellow powder was dissolved in 500 μL ethanol (95%) and the amount of callus peaks was evaluated by HPLC equivalent to dose 284 μg/plate, 1870 μg/plate of silymarin SIGMA and 226 μg/plate of seed extract. Then 50 μL of the stock culture of *S. typhimurium* TA98 was inoculated in 5 mL of Nutrient Broth medium including 5 μL ampicillin (40 mg/mL), 0.96 μL histidine (26 mM) and 0.5 mL biotin (0.5 mM). The mixture incubated with shaking at 37 °C for 16–18 h. Minimal-Agar plates were supplemented with 25× VBMM (Vogel-Bonner minimal medium) solution, 40% glucose and 40 μg/mL ampicillin. Before use, the plates were treated with 100 μL of 0.5 mM biotin. Agar-agar (6 g/L) dissolved in 1 mL distilled water was heated with 5 g/L NaCl after cooling to about 50 °C, 5 mL of a 0.5 mM histidine/biotin stock solution was added to 50 mL. The Ames test was prepared in 15 mL Greiner tubes containing 400 μL PBS, pH 7.5, mixed with 100 μL *S. typhimurium* working culture and 50 μL ± test compound (Silymarin or callus extract or seed extract). The culture was incubated at 37 °C for 1 h. After that, a 2 μL genotoxic compound NPD for mutation or 2 μL ethanol as negative control determination were added and incubated for 5 min. The mixture with 2 mL top agar was poured onto agar plates which were incubated at 37 °C for 48 h. After removing the plates from the incubator, colonies were counted with an electronic counter. The results are expressed as revertant colonies per plate. Data derived from six independent experiments. 

### 2.9. Statistical Analysis 

The obtained results were statistically assessed for the significance degree of variations using one and two way ANOVA [41]. All statistical methods were applied using the SPSS statistical software package. 

## 3. Results and Discussion

### 3.1. Callus Culture and Production of Flavonoids, Phenolics, and Saponins 

Calli of *S. marianum* grown in media A to O supplemented with different growth regulators [42] (Table 1) were analyzed for contents of the secondary metabolites saponins, phenolics, and flavonoids. Results (Table 2) depicts that the metabolite contents of the callus tentatively were in the order saponins > phenolics > flavonoids with means 36.3, 9.0, and 2.8 mg/g DW, respectively. This quantitative comparison should be taken with caution, because the metabolite groups were determined relative to standard reference compounds and, due to the diverse nature of similarly reacting compounds, could not be determined in absolute quantities by different methods. The three groups of compounds displayed the same pattern in the seeds of *S. marianum* but with significantly lower values (12.0, 6.0, and 0.6 mg/g DW respectively). However, callus produced 3.0-, 1.5-, and 4.6- times more saponins, phenolics, and flavonoids, respectively relative to the plant seeds. The most pronounced difference was in the flavonoids contents which correlate with the importance of *S. marianum* in treating liver diseases, including liver cirrhosis. The low concentrations of 2,4-D (0.25 mg/mL) combined with intermediate concentrations of Asn (50 mg/L), BAP (0.05 mg/L), and inositol (50 mg/L) as in media F produced the highest amount of flavonoids and phenolic compounds (4.7 and 16.0 mg/g respectively). In a converse manner, calli grown in intermediate 2,4-D concentrations (0.5 mg/mL) in the absence of Asn, BAP, and inositol (media H) accumulated the lowest amounts of flavonoids and phenolic compounds (0.7 and 3.0 mg/g respectively). 

The best combination of growth regulators increased the contents of the two secondary compounds 5.3 and 6.7-times compared to the lowest contents. Callus growing in the presence of 0.5 mg/L 2,4-D with Asn (50 mg/L), BAP (0.05 mg/L) and inositol (50 mg/L) (media J) accumulated high amounts of saponins (53.0 mg/g DW). However the saponins compounds were absent in *Silybum marianum* plant [20] but their presence was recorded for seeds with 12 mg/g DW indicating that, the callus shows high potential for saponins accumulation that may increase its qualitative value for medical purposes. In a converse manner, 0.25 mg/L 2,4-D in the absence of Asn, BAP, and inositol (media D) produced the lowest amount of saponins (24.0 mg/g DW) that was double of the plant seeds. The total amounts of flavonoids, phenolic compounds, and saponins in *S. marianum* callus (48.1 mg/g DW) was 2.6-times (18.6 mg/g DW) that of seeds. However, accumulation of secondary metabolites in seeds was much lower than in all media calli grown particular, media F, I, and J.

Results revealed that *S. marianum* callus may serve as an alternative source for liver-protective pharmacological drugs and thus callus-based approaches would be of significant interest for economic and ecological reasons. The callus induction from cotyledons was strongly affected by endogenous and exogenous factors as represented in the present study. Medium supplementation with mineral elements, growth regulators, amino acids and vitamins is known to influence callus induction and growth rate in addition to its potential for secondary metabolites accumulation [43,44,45]. The use of 2,4-D alone or in combination with amino acids or other hormones has become a common approach in promoting cell culture or callus formation and inducing somatic embryogenesis in seed culture [46,47,48].

### 3.2. HPLC and Mass Spectrometric Analysis 

**Figure 1:**
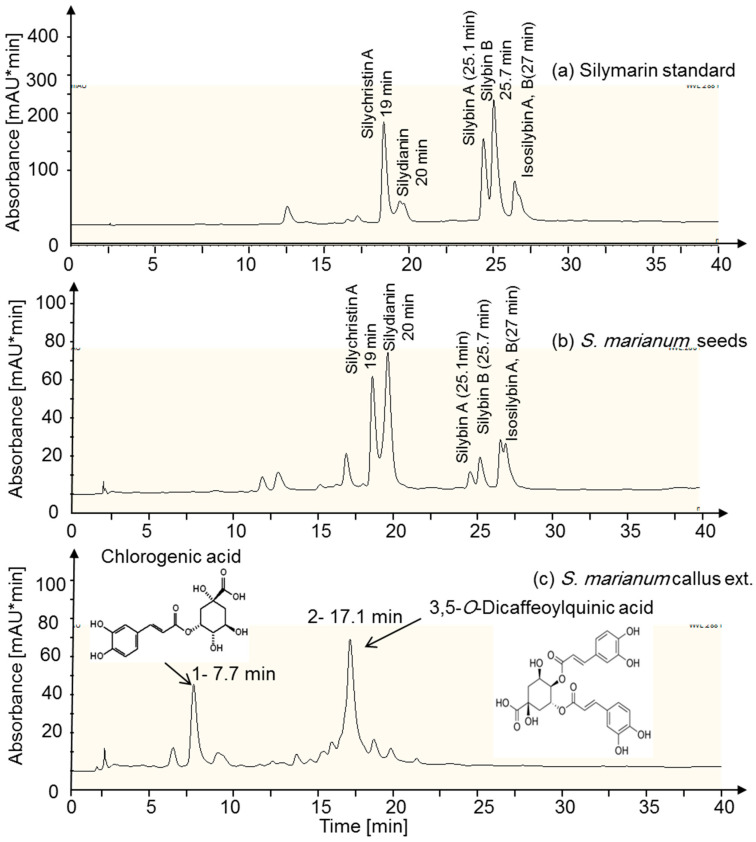
HPLC chromatograms of methanolic extracts. (**a**) Standard silymarin (SIGMA), (**b**) Silymarin from *Silybum marianum* seeds of the Egyptian cultivar, and (**c**) *S. marianum* callus derived from cotyledons. Absorbance of fractions was monitored at 288 nm. The separation was performed ≥ three times with similar result.

The nature of the compounds, extracted from callus and seeds as well as standard silymarin, was identified by HPLC separation followed by mass spectrometry. Mostly similar peaks were revealed for standard silymarin and seeds extract (Figure 1A,B). Silybin A, silybin B, isosilybin A, isosilybin B, silydianin, and silychristin are the main constituents and silybin A and B are considered as biologically most active components among the silymarin constituents [49]. The compounds were identified by their retention time and mass at 481 m/z are in line with the reported data [50]. The peaks area indicated that the used Egyptian ecotype *Silybum marianum* with purple flower contained low amounts of silybin A and B compared to standard silymarin.

The callus extracts separated into different peaks with two dominant ones in the UV-spectrum of callus that were missing in standard silymarin and seed extract. The first peak eluted after 7.7 min in the HPLC chromatogram (Figure 1C).

**Figure 2:**
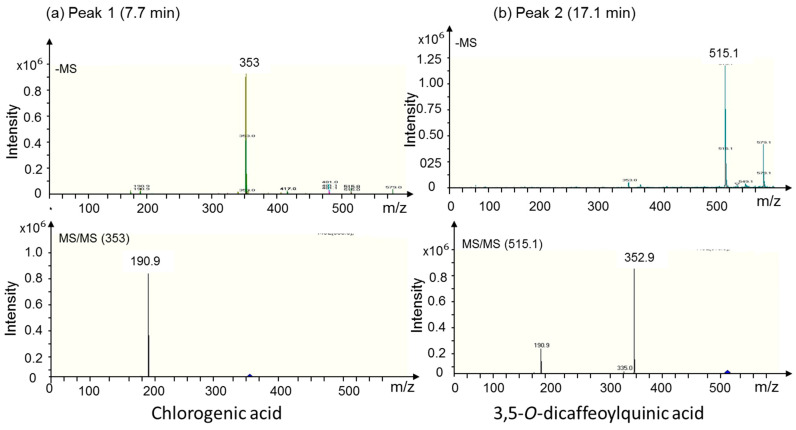
Fragmentation pattern of the peaks 1 (7.7 min) and 2 (17.1 min). (**a**) ESI-MS and MS/MS in the negative mode of the ion for peak 1 (7.7 min). The obtained m/z values reflect the molecular weight of chlorogenic acid (353). (**b**) ESI-MS and MS/MS (negative mode) of the ion for peak 2 (17.1 min). The obtained m/z value represent the molecular weight of 3,5-*O*-dicaffeoylquinic acid (515).

**Figure 3:**
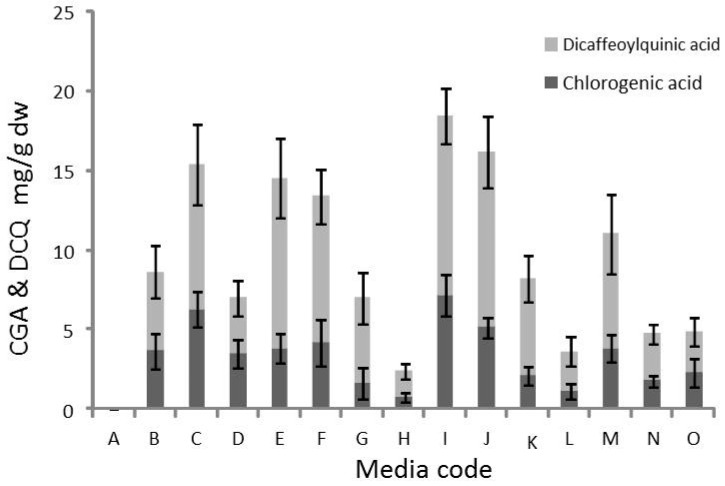
Quantitative HPLC analysis. Chlorogenic acid (height of dark grey bars) and 3,5-O-dicaffeoylquinic acid (light grey bars) were quantified in methanolic extract of *S. marianum* callus. Data are means from three independent experiments ± SE.

ESI-MS and MS/MS identified the compound as chlorogenic acid (CGA), based on its mass of 353 m/z and the fragment mass of 190.9 m/z in the MS/MS-mode (Figure 2). Both masses are in line with the reported data [51]. The second dominating peak eluted after 17.1 min and revealed an ESI-MS mass of 515.1 m/z and a main MS/MS fragment of 352.9 m/z (Figure 1 and Figure 2) which corresponds to the molecular mass of 3,5-*O*- dicaffeoylquinic acid (DCQ). Both masses matched with reported ones [52]. CGA and DCQ were quantified from peak areas by comparison with standard CGA and DCQ (Table 3), thus providing information on slightly higher yield of DCQ than of CGA in the callus in response to all medium growth regulators (media A to O). The data also show that medium I with medium concentrations of 2,4-D and low concentrations of Asn, BAP and, inositol produced the highest yield of both CGA and DCQ. Medium J produced the second highest yield, while medium H with medium concentrations of 2,4-D but lacking Asn, BAP and inositol produced very small amounts of the two acids. A variation of CGA and DCQ production by 29% (average of all results) was observed. The variation decreased with higher yield, e.g., in callus from medium I (16% and 18%, respectively). Such fluctuations in yield may indicate instability of production. It is worth mention that callus from all media displayed the same chromatographic pattern, but with different areas reflecting the metabolism of the different compounds in response to the applied growth regulators (Figure 3).

The results also revealed that none of the callus growth regulator combination supported production and transformation of flavonoids to silymarin fractions. Instead the callus synthesized chlorogenic acid and 3,5-O-dicaffeoylquinic acid. The accumulation of chlorogenic acid and 3,5-O-dicaffeoylquinic acid may indicate that the callus fails to express the metabolic genes needed for the conversion of both compounds in to silymarin. Cell cultures of *S. marianum* was also found to produce phenolic acids such as chlorogenic acid, isochlorogenic acid A, B, and C, trans-ferulic acid, 4-methoxy-cinnamic acid, and vanillic acid [7].

Chlorogenic acid is a precursor produced both in cells and callus cultures of *S. marianum*, in addition to cynarin. 3,5-O-dicaffeoylquinic acid is a double ester of caffeic acid. Quinic acid was produced only in the plant callus as a predominant phenolic compound. Caffeic acid is also synthesized by shikimic and phenylpropanoid pathway but take a different branch than flavonoids. The p-Coumaroyl-CoA can enter the flavonoid biosynthesis or alternatively can be converted to caffeoyl-CoA and coupled with quinic acid to form 5-O-caffeoylquinic acid [53].

The ability of calli grown from *S. marianum* cotyledons to accumulate CGA and CQA to high amounts is of pharmaceutically interesting since they are known for their positive effects on liver function. Likewise, chlorogenic acid exhibits a better therapeutic protective action than silymarin in CCl_4_ (carbon tetrachloride)-treated mice [54]. Isochlorogenic acids significantly improve the viability of hepatocytes at concentrations of 1 to 100 μg/mL that afforded much stronger liver protection than the reference drug silybinin at 100 μg/mL concentration [55]. 

Interestingly, the callus biomass production did not reveal the expected negative correlation with phenolics including CGA+DCQ accumulation, but rather a slightly positive dependency was remarked. Often synthesis of defense compounds negatively affects biomass production as tradeoff, which is not observed in the present study. However, medium I produced the greatest callus weight [42] as well as the highest contents of CGA+DCQ. Thus, the well-growing callus conditions tended to produce slightly more CGA+DCQ. 

Only detailed HPLC analyses coupled with mass spectrometric identification is reliable for the compounds of silymarin detection as illustrated in the present study. The assay for silymarin compounds based on 2,4-dinitrophenylhydrazine which reacts with carbonyl groups of ketones and aldehydes, is not reliable for silymarin detection, as proved by our previous study which estimated such phenolic compounds spectrophotometrically and considered it as silymarin [42]. Thus, despite the fact that it has been repeatedly used to spectrophotometrically assess silymarin contents of *S. marianum* tissues and extracts [56], such data must be considered with care. This study confirms that phenolic compounds such as chlorogenic acid and dicaffeoylquinic acid are also react with the reagent 2,4-dinitrophenylhydrazine. 

### 3.3. Anti-Genotoxicity Effects of Callus Extract 

**Figure 4:**
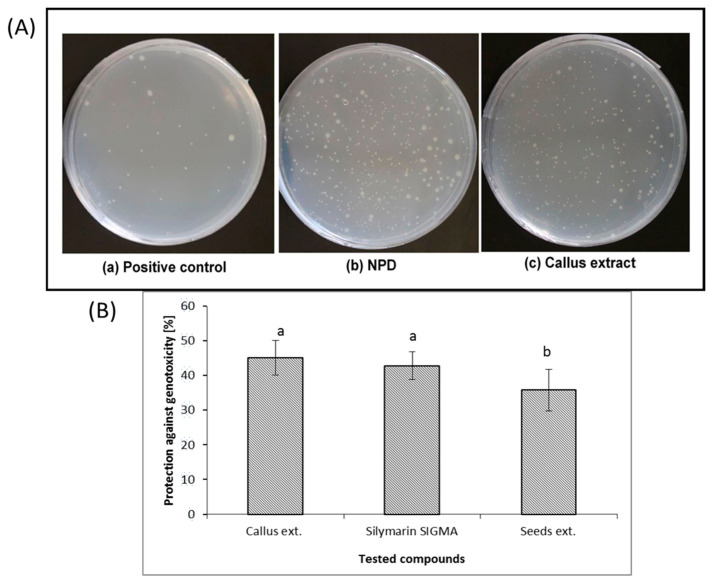
(**A**) Effect of callus extract on 4-nitro-*o*-phenylenediamine (NPD)-induced genotoxicity in *S. typhimurium* TA98 strain. (**a**) Spontaneously occurring revertants (–his media). (**b**) A high number of colony revertants in presence of NPD. (**c**) Lower number of colony revertants in NPD-treated TA98 in the presence of callus from *Silybum marianum* cotyledons. (**B**) Protection percentage of tested compounds (Callus extract, Silymarin SIGMA, and seed extract) against genotoxicity induced by 4-nitro-o-phenyldiamine (NPD). Callus extract, Silymarin SIGMA, and seed extract reduced genotoxicity of NPD by 45.2%, 42.8%, and 35.8% respectively. Data are means±SE from six independent experiments.

The AMES assay was employed to assess the protection against a standard mutagen in *Salmonella typhimurium* TA 98, OPD, of callus extracts in comparison with that of standard silymarin and *S. marianum* seed extract (Figure 4). Figure 4 shows that callus extract recorded the highest protection against NPD of 45.2% at a dose of 284 μg/plate in comparison with standard silymarin that reduced the genotoxicity of the NPD by 42.8% at a higher dose of (1870 μg/plate) (normalized to chlorogenic acid) as determined by the decreasing number of His+ revertants. Seed extract caused the least effect on NPD (by 35.8% at a dose of 226 μg/plate).

The *S. marianum* callus extract strongly decreased the rate of reverse mutations induced by *S. typhimurium* TA98 by 45.2% at dose 284 μg/plate. This indicate that the recorded compounds in the callus extract (CGA and DCQ) acquired a greater effect than standard and silymarin components from seeds. Caffeic acid and chlorogenic acid were found to suppress the mutagenic action of N-methyl-N-nitro-nitrosoguanidine in *S. typhimurium* strain TA 1535 by scavenging electrophilic degradation products of the cancer-causing agents [57,58]. In this regard, Silymarin at dose 1000 μg/plate reduced the genotoxicity of DMH, AOM and MNU agents in a dose-dependent manner and the number of His+ revertants per plate decreased by 50% at the highest dose of silymarin [59]. Both intracellular and extracellular systems contribute to the activity of phenolics against mutagens [60,61].

## 4. Conclusions

Media optimization through different concentrations of growth regulators 2,4-D, benzylaminopurine, myoinositol, and asparagine failed to support the production of flavonlignans of the complex silymarin production in the cells. However, instead the callus synthesized chlorogenic acid and 3,5-O-dicaffeoylquinic acid that have anti-genotoxic activity that suppresses the mutagenic action of 4-nitro-o-phenylenediamine in *Salmonella typhimurium* TA98. This study provides the experimental framework for the bioproduction of the two medicinal compounds from *Silybum marianum* cotyledon callus.

## Figures and Tables

**Table 1:** Experimental design and composition of callus growth media A to O. The table gives the levels of 2,4-D, asparagines, benzyladenine, and myo-inositol (mg/L).

2,4-D (mg/L)	Asparagine (mg/L)				Benzyladenine (mg/L)				Myo-Inositol (mg/L)			
	0.0	25	50	100	0.0	0.01	0.05	0.1	0.0	25	50	100
0.0	0	A	B	C	0	A	B	C	0	A	B	C
0.25	D	E	F	G	D	E	F	G	D	E	F	G
0.5	H	I	J	K	H	I	J	K	H	I	J	K
1.0	L	M	N	O	L	M	N	O	L	M	N	O

**Table 2:** Effect of medium modifications on callus contents of flavonoids, phenolic compounds and saponins in comparison with the seeds’ contents. Each value represents the mean ± SE from six replicates. NI: No callus induction.

	Growth Regulators Level (mg/L)				Flavonoids	Phenolic Compounds	Saponin	Total
Media Code	2,4-D	Asn	BAP	Inositol	(mg/g dw)			
A	0	25	0.01	25	NI	NI	NI	NI
B	0	50	0.05	50	1.4 ± 0.2 ^c^	5 ± 0.6 ^bc^	33 ± 1 ^ab^	39.4
C	0	100	0.1	100	4.2 ± 0.2 ^fg^	13 ± 0.9 ^hi^	32 ± 2 ^ab^	49.2
D	0.25	0	0	0	1.6 ± 0.2 ^c^	4 ± 0.7 ^ab^	24 ± 2 ^ab^	29.6
E	0.25	25	0.01	25	4.4 ± 0.3 ^g^	14 ± 0.3 ^i^	33 ± 2 ^ab^	51.4
F	0.25	50	0.05	50	4.7 ± 0.3 ^g^	16 ± 0.8 ^j^	42 ± 5 ^b^	62.7
G	0.25	100	0.1	100	2.6 ± 0.1 ^d^	10 ± 1 ^g^	33 ± 2 ^ab^	35.6
H	0.5	0	0	0	0.7 ± 0.03 ^ab^	3 ± 0.7 ^a^	26 ± 1 ^ab^	29.7
I	0.5	25	0.01	25	4.2 ± 0.4 ^fg^	12 ± 2.2 ^h^	32 ± 1 ^ab^	48.2
J	0.5	50	0.05	50	3.8 ± 0.2 ^ef^	12 ± 1.1 ^gh^	53 ± 9 ^e^	68.8
K	0.5	100	0.1	100	3.4 ± 0.1 ^e^	10 ± 1.6 ^g^	52 ± 16 ^e^	65.4
L	1	0	0	0	1.7 ± 0.03 ^c^	4 ± 1.4 ^ab^	27 ± 7 ^c^	31.0
M	1	25	0.01	25	2.4 ± 0.1 ^d^	9 ± 1.2 ^f^	43 ± 4 ^d^	54.4
N	1	50	0.05	50	1.3 ± 0.2 ^bc^	8 ± 0.5 ^ef^	46 ± 20 ^d^	55.3
O	1	100	0.1	100	1.2 ± 0.2 ^bc^	6 ± 0.7 ^cd^	42 ± 2 ^b^	49.2
Mean content in callus					2.8 ± 1.5	9.0 ± 2.1	36.3 ± 2	48.1
Mean content in seeds					0.6 ± 0.03	6 ± 0.5	12 ± 4	18.6
F-value					50.24	60.27	171.3	
Probability *p*<					0.00	0.00	0.00	

Means in the same column have similar letter are significantly varied at *p* < 0.05.

**Table 3:** Chlorogenic acid and dicaffeoylquinic acid contents of the two major peaks from *S. marianum* callus. Each value represents the mean ± SE from three replicates NI: No induction.

	Peak 1: MS^1^ 353, MS/MS 190.1		Peak 2: MS^1^ 515.1, MS/MS 352.9	
Media Code	T_R_ (min)	Chlorogenic Acid (mg/g DW)	T_R_ (min)	Dicaffeoylquinic Acid (mg/g DW)
A	NI	NI	NI	NI
B	7.9	3.7 ± 1.1 ^bcde^	17.4	5.0 ± 1.6 ^abc^
C	7.9	6.3 ± 1.1 ^ef^	17.4	9.1 ± 2.5 ^cde^
D	7.9	3.5 ± 0.9 ^bcd^	17.2	3.5 ± 1.1 ^ab^
E	7.8	3.9 ± 1.0 ^cde^	17.2	10.7 ± 2.5 ^de^
F	7.8	4.2 ± 1.4 ^cde^	17.2	9.2 ± 1.7 ^cde^
G	7.8	1.7 ± 1.0 ^abc^	17.2	5.4 ± 1.6 ^abc^
H	7.7	0.8 ± 0.3 ^a^	17.1	1.7 ± 0.5 ^a^
I	7.7	7.2 ± 1.3 ^f^	17.1	11.3 ± 1.8 ^e^
J	7.7	5.2 ± 0.6 ^def^	17.1	6.1 ± 2.3 ^e^
K	7.7	2.2 ± 0.6 ^abc^	17.1	6.0 ± 1.4 ^abcd^
L	7.7	1.1 ± 0.5 ^ab^	17.1	2.5 ± 1.0 ^ab^
M	7.7	3.8 ± 0.8 ^cde^	17.0	7.2 ± 2.5 ^bcde^
N	7.7	1.8 ± 0.4 ^abc^	17.0	2.9 ± 0.6 ^ab^
O	7.7	2.4 ± 0.9 ^abc^	17.0	2.5 ± 0.9 ^ab^
Mean	7.8	3.41	17.2	5.94

Means in the same column have similar letter are significantly varied at *p* < 0.05.
